# Cost-effectiveness of Catheter Ablation Versus Antiarrhythmic Drug Therapy for the Treatment of Atrial Fibrillation: A Canadian Perspective

**DOI:** 10.36469/9837

**Published:** 2016-10-26

**Authors:** Yaariv Khaykin, Peter J. Mallow, John A. Rizzo, Atul Verma, Lauren Chun, Shelby Olesovsky, Matthew R. Reynolds

**Affiliations:** 1 Southlake Regional Health Centre, Newmarket, Ontario, Canada; 2 CTI Clinical Trial and Consulting Services, Inc., Cincinnati, OH, USA; 3 Stony Brook University, Stony Brook, NY, USA; 4 Lahey Hospital & Medical Center, Burlington, MA, USA

**Keywords:** markov model, cost-utility, canada, atrial fibrillation, cost-effectiveness

## Abstract

**Background:** Atrial fibrillation (AF) affects approximately 350,000 Canadians and has an estimated annual economic burden exceeding $800 million dollars. Anti-arrhythmic drug (AAD) therapy and catheter ablation (CA) are the two common treatments for paroxysmal AF. However, the upfront costs of CA are quite substantial.

**Objective:** The objective of this study was to assess the cost-effectiveness of CA compared to AAD for AF based on community practice.

**Methods:** A Markov simulation model was developed for a hypothetical cohort of 55-year-old patients with paroxysmal AF and a low stroke risk. Patients received either CA or AAD. Costs and quality-adjusted life years (QALYs) were computed over lifetime, 10-year, and 5-year time horizons. Model inputs were obtained from a large, prospectively collected, single-center Canadian registry and augmented with the published literature, using Canadian cost estimates for disease states. Threshold values of $25,000, $50,000, and $100,000 per QALY, respectively, were used to determine cost-effectiveness. All costs were expressed in 2012 Canadian dollars.

**Results:** The incremental cost-effectiveness ratio for CA versus AAD therapy was $1,228, $22,879, and $63,647 for the lifetime, 10-year, and 5-year time horizons, respectively. Over a lifetime horizon, the probability of achieving cost-effectiveness was 100% for all 3 cost per QALY thresholds. The 10-year probability of achieving cost-effectiveness was 74%, 100%, and 100% at the $25,000, $50,000, and $100,000 thresholds, respectively. The 5-year probability of achieving cost-effectiveness was 0%, 0.9%, and 100% at the 3 cost per QALY thresholds. Results were most sensitive to time horizon, probability of repeat AF ablation, and stroke rate.

**Conclusions:** From the perspective of the Canadian Healthcare system, CA is a potentially cost-effective treatment compared to AAD therapy in a low stroke risk population using real-world data when examining a time horizon of greater than 5 years.

## INTRODUCTION

Approximately 350,000 Canadians are currently affected by atrial fibrillation (AF).[1] This condition is characterized by disorganized, rapid, and irregular heartbeat. Patients with AF are at increased risk of subsequent stroke, morbidity, and mortality.[2-4] The rate of hospitalization for AF in Canada was approximately 583 per 100,000 people between 1997 and 2000, with an average of 129,000 hospitalizations per year.[5] As a result, the aggregate economic burden of AF, including initial costs as well as over the longer term, are quite substantial. A recent study examining the hospital costs associated with AF found the burden to exceed $800 million (2010 Canadian Dollars).[6]

The significant burden to the health care system presented by AF has sparked interest in seeking better ways to manage AF. Proper AF management is a multi-fold approach, which seeks to control the heart rate, prevent thromboembolism, and correct the rhythm disturbance.[7] Antiarrhythmic drug (AAD) therapy is commonly used to treat paroxysmal AF, but it has had mixed results.[8] While these drugs are used to help restore and maintain normal sinus rhythm (NSR), limitations include inconsistent efficacy and frequent side effects. As a result, non-pharmacological approaches such as catheter ablation (CA) are becoming increasingly popular treatment alternatives.[9,10] CA has been shown to be more effective and to improve quality of life in paroxysmal AF patients who have failed an AAD therapy compared to those continuing with AAD therapies.[8, 11]

While a number of studies have performed economic evaluations comparing CA to AAD, there is no clear consensus on whether CA is a cost-effective treatment option.[12] One important limitation in previous research is the lack of real-world data pertaining to CA[8,12], which adds to uncertainty about the economic value of this treatment alternative in actual practice. The purpose of this study was to assess the cost-effectiveness of CA compared to AAD therapy using real-world outcomes data from the perspective of the Canadian Healthcare system.

## METHODS

### Study Population and Perspective

At the time of intervention, the population was assumed to be 55 years of age with paroxysmal AF and to be at low risk of incurring a stroke, i.e. $CHADS_2$ score of 0 or 1. The published literature suggests that most ablations occur in individuals under the age of 60, and the registry data used in the analysis was consistent with the literature.[11] These patients were followed over lifetime, 10-year and 5-year periods. The analysis is from a government payer perspective e.g., the Ontario Ministry of Health.

### Model Structure and Treatment Strategies

A Markov model was used to simulate the transitions between health states throughout a patient's lifetime. Cycle times in the model were 6 months and Monte Carlo Simulation was performed. Patients were treated either with CA or AAD. The cost-utility analysis included costs, quality-adjusted life years (QALYs) for each health state, and Incremental Cost-Effectiveness Ratios (ICERs) for both treatment arms. Threshold levels of $25,000, $50,000, and $100,000 per QALY were used to assess cost-effectiveness. A 5% discount rate was applied to all costs and QALYs. All costs were expressed in 2012 Canadian dollars.

In the ablation treatment arm, patients were assumed to have failed a first-line AAD therapy (i.e. sotalol; AAD1) and receive their first ablation at the start of the model. Patients with NSR who had an AF recurrence received another ablation. The model assumed that patients may have a maximum of three ablations and that the procedural complication rate was the same for each ablation. Patients who had a successful ablation may still receive oral anticoagulant (OAC) therapy. Procedural complications included: pulmonary vein (PV) stenosis, tamponade, transient ischemic attack (TIA), stroke and death. Patients for whom a third ablation treatment was unsuccessful receive a second-line AAD therapy (i.e. amiodarone; AAD2). If the patient failed while on second-line AAD therapy, then the patient received rate control medications alone. Figure 1 illustrates treatment patterns and outcomes in the ablation treatment arm.

Patients in the AAD treatment arm are assumed to have failed a first-line AAD therapy (AAD1) and are provided with another first-line AAD therapy. Those patients who fail on this new first-line AAD therapy are then moved to second-line AAD therapy (AAD2). The model assumes that patients in the AAD arm cannot crossover to ablation. This assumption better delineated differences in CA and AAD treatment. Patients who fail on the second-line AAD therapy will be provided rate control medication only. Treatment patterns and outcomes for the AAD arm are described in Figure 2.

**Figure 1.** Ablation Arm Diagram

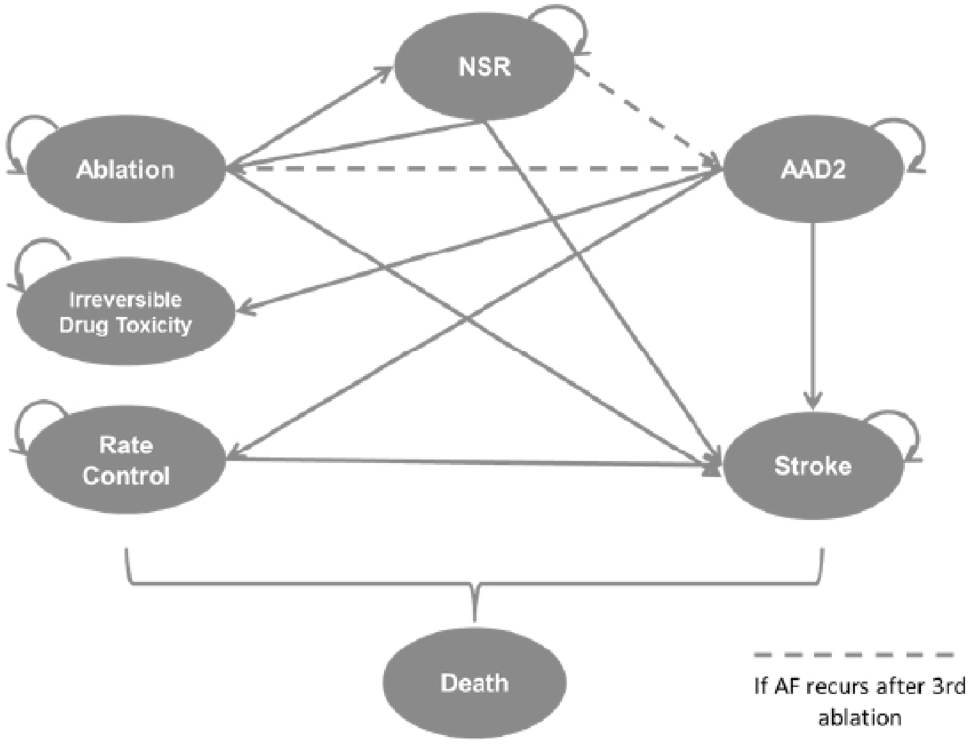

AAD2: Second-line AAD therapy (i.e. amiodarone); NSR: Normal sinus rhythm

This simplified diagram illustrates the major health states (ovals) and possible transitions between health states (arrows) for a patient in the ablation treatment strategy. Patients begin in the ablation health state and can transition to the next health (i.e. NSR) state or remain in the current health state based on the arrows and transition probabilities (see Online Resource Table 1). There is a maximum of three ablation procedures permitted in the model. Each health state has a background mortality risk based on age and can transition to death.

**Figure 2.** AAD Arm Diagram

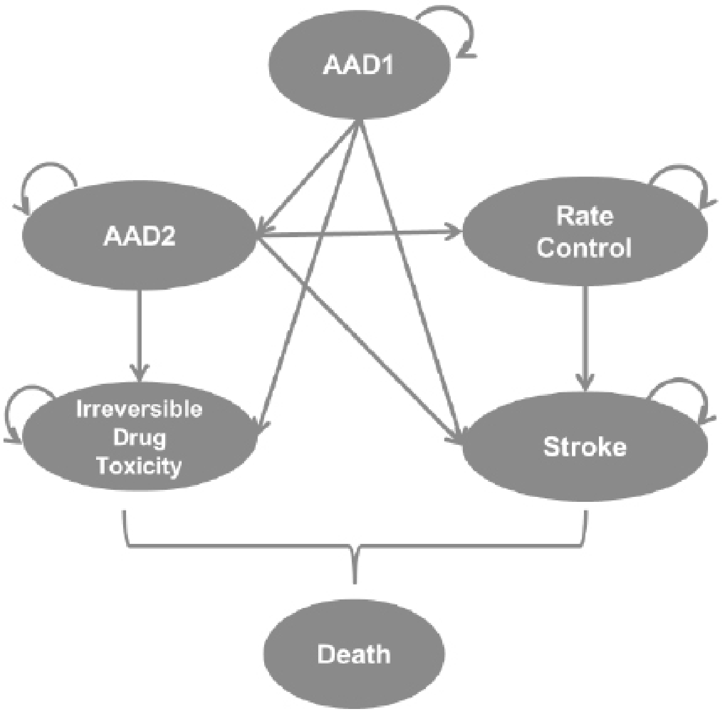

AAD1: first-line AAD therapy (i.e. sotalol); AAD2: second-line AAD therapy (i.e. amiodarone)

This simplified diagram illustrates the major health states (ovals) and possible transitions between health states (arrows) for a patient in the AAD treatment strategy. Patients begin in the AAD1 health state and can transition to the next health state or remain in the current health state based on the arrows and transition probabilities (see Online Resource Table 1). Each health state has a background mortality risk based on age and can transition to death.

There were several model characteristics common to both treatment arms. Health state utility values were derived from the published literature for each health state. Patients who received OAC therapy received a new OAC drug such as dabigatran rather than warfarin.[13] Because OAC therapy is common to both treatment arms, assuming warfarin therapy rather than dabigatran would not change the results qualitatively. The choice of first- and second-line AAD therapy was informed by the 2012 Canadian Cardiovascular Society Guidelines.[13] Non-fatal drug toxicity was included as a complication resulting from AAD therapy. Patients had a background stroke risk based on the literature that depended on age and whether or not they were in AF or NSR. Strokes would lead to either mild disability, moderate-to-severe disability, or death. Finally, patients in all health states could transition to death.

### Data Sources

Clinical data were prospectively collected from a single center Canadian registry at the Southlake Regional Health Center in Ontario (Southlake Database). Procedural and patient data have been described previously.[14-17] This database included 559 patients who underwent CA from 2004 to 2013. The average follow up time for all patients was 18.2 months. The Southlake database has been approved by the Southlake Regional Health Center Institutional Ethics Review Board. Additional clinical data were augmented as necessary from the published literature. Cost data were used from the perspective of the Canadian Healthcare system.

Health state utility values from the published literature were used to help define QALYs. Patients in NSR had a significantly higher health state utility value than those in an AF or other health state. Mortality rates were derived from Canadian life table data. All model parameter values, ranges, distributions, and sources are described in Online Resource Table 1.

### Sensitivity Analysis

One-way sensitivity analyses were performed to gauge the robustness and reliability of the results. The sensitivity analyses entailed varying key model input values one-by-one and recalculating the cost-effectiveness results each time a change was made. Important changes investigated in the sensitivity analysis included: discount rate (0%, 3%, and 8%); time period (10-year, 5-year); starting age; stroke rate assumptions between NSR and AF; and health state utility assumptions between NSR and AF. We also performed probabilistic sensitivity analysis, recalculating ICERs using probabilistic distributions for model parameters and Monte Carlo simulation methods. This allowed for scenarios that consider more simultaneous variations in model values than does the one-way sensitivity analysis.

### Model Validation

The model was validated in two ways. First, rates of repeat ablations from the model were compared to actual repeat ablation rates from the Southlake Regional Health Center. Second, the results of the economic evaluation were compared to those in the Canadian Agency for Drugs and Technologies in Health (CADTH) technology report.[8]

## RESULTS

Table 1 shows the results for the cost-utility analysis, which assumed lifetime follow-up as well as shorter time horizons. In the base case model assuming a lifetime horizon, CA is expected to have an ICER of $1,228 and be cost-effective 100% of the time at willingness-to-pay thresholds of $25,000; $50,000; and $100,000, respectively. Examining a 10-year time horizon, CA is expected to have an ICER of $22,879 and be cost-effective 74%; 100%, and 100% at willingness-to-pay thresholds of $25,000; $50,000; and $100,000, respectively. When a 5-year time horizon is considered, CA is expected to have an ICER of $63,647 and be cost-effective 0.0%; 0.9%, and 100% at willingness-to-pay thresholds of $25,000; $50,000; and $100,000, respectively.

**Table 1.** Cost-utility Analysis (CUA) Results for Lifetime, 10-year and 5-year Time Horizons – Start Age 55

| | Strategy | Mean Costs[a] | Mean QALYs | ICER | Probability of Cost-Effectiveness at $25,000/QALY | Probability of Cost-Effectiveness at $50,000/QALY | Probability of Cost-Effectiveness at $100,000/QALY |
|---|---|---|---|---|---|---|---|
| **Lifetime** | Ablation | $39,956 | 10.49 | $1,228 | 100.0% | 100.0% | 100.0% |
| | AAD | $37,955 | 8.86 | | | | |
| **10-year** | Ablation | $24,142 | 5.49 | $22,879 | 73.8% | 100.0% | 100.0% |
| | AAD | $12,261 | 4.97 | | | | |
| **5-year** | Ablation | $19,499 | 2.96 | $63,647 | 0.0% | 0.9% | 100.0% |
| | AAD | $6,182 | 2.75 | | | | |

[a]Costs are reported in 2012 Canadian Dollars discounted at 5%; AAD: Antiarrhythmic drug therapy; ICER: Incremental cost-effectiveness ratio; QALYs: Quality-adjusted life years

Table 1: The mean costs are the average expected costs incurred by a patient in the respective treatment arm. The mean QALYs are the expected number of quality-adjusted life years for a patient in the respective treatment arm. The ICERs are determined by subtracting the difference in costs between Ablation and AAD and dividing by the difference in QALYs between Ablation and AAD. The probability of cost-effectiveness represents the percentage of model iterations that resulted in an ICER less than the stated threshold.

### Sensitivity Analysis

As a first robustness check, the analysis was repeated assuming that the study population is 65 years of age instead of 55 years. The results, summarized in Online Resource Table 2, are quite consistent with the base case findings. Assuming a lifetime follow-up, CA is a dominant treatment strategy. That is, it leads to lower costs and more QALYs. Assuming a 10-year time horizon, CA has an ICER of $16,861 and is cost-effective 98.9%, 100% and 100% at willingness-to-pay thresholds of $25,000, $50,000 and $100,000, respectively. When a 5-year time horizon is considered, CA is expected to have an ICER of $55,799 and be cost-effective 0.0%; 16.6%, and 100% at willingness-to-pay thresholds of $25,000; $50,000; and $100,000, respectively.

Variations in the discount rate, health state utility from AF, the probability of reversion back to AF following ablation, and risk of stroke were considered as further robustness checks. These results, summarized in Table 2, confirmed that CA remains an attractive alternative to AAD when these model parameter values change.

**Table 2.** Cost-utility Analysis (CUA) Results – Alternative Model Scenarios

| | Incremental Costs[a] (Ablation vs. AAD) | Incremental QALYs (Ablation vs. AAD) | ICER |
|---|---|---|---|
| **Discount Rate** | | | |
| 0% | -$17,683 | 3.8 | Dominant[b] |
| 3% | -$2,958 | 2.23 | Dominant[b] |
| 8% | $6,471 | 1.1 | $5,874 |
| **Utility Reduction for Atrial Fibrillation** | | | |
| No utility reduction for AF | $2,045 | 1.4 | $1,464 |
| **Annual Probability of Reversion back to Atrial Fibrillation after Ablation** | | | |
| 5% | $4,665 | 1.58 | $2,950 |
| 10% | $11,736 | 1.37 | $8,549 |
| **Annual Probability of Stroke** | | | |
| No difference between NSR & AF stroke rate | $14,022 | 0.87 | $16,172 |
| Recurrent stroke rate set to 0 | $5,975 | 1.55 | $3,867 |

[a]Costs are reported in 2012 Canadian Dollars discounted at 5%; [b]A dominant strategy costs less and increases effectiveness; ICER: Incremental cost-effectiveness ratio; NSR: Normal sinus rhythm; QALYs: Quality-adjusted life years

Table 2 illustrates different scenarios of the lifetime model comparing Ablation to AAD by changing key model assumptions. The ICERs are determined by dividing the incremental costs by the incremental QALYS.

Changes in various cost and utility parameter values are also included as part of the sensitivity analysis. These results are summarized in Figures 3 through 5 for costs, health state utilities, and ablation complication rates, respectively. As the figures reveal, CA remains cost-effective under a variety of alternative cost, health state utility and complication rate scenarios. The one-way sensitivity analyses reveal that the results were most sensitive to the time horizon, probability of repeat AF ablation, difference in stroke rate between NSR

and AF, and the discount rate.

As a final check on robustness, a probabilistic analysis was conducted via Monte Carlo simulation methods. The results, shown in Online Resource Figure 1, confirm that the ICER for CA remains well below the threshold willingness-to-pay values.

**Figure 3.** One-way Sensitivity Analysis - Costs

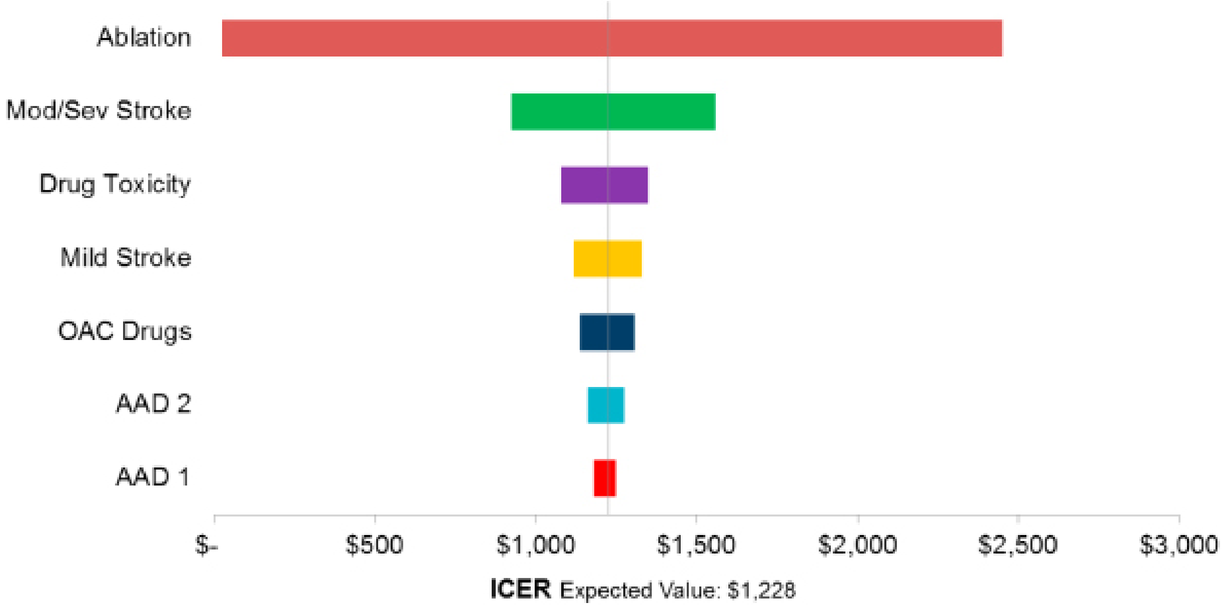

AAD 1: First-line anti-arrhythmic drug therapy; AAD 2: Second-line anti-arrhythmic drug therapy; ICER: Incremental cost-effectiveness ratio; NSR: Normal sinus rhythm

The tornado diagram displays the results of one-way sensitivity analyses for key cost parameters on the ICER for Ablation compared to AAD. The base case ICER is denoted by the vertical line, and changes in the ICER for each parameter are denoted by the horizontal bar.

## Model Validation

The model's repeat ablation rates were quite consistent with actual rates from the Southlake Regional Health Center. In particular, the model specifies that repeat ablation rates within the first year of ablation were 19.4%, quite close to the 18.3% rate from Southlake. Similarly, the rate of third ablation within one year of the second ablation was 2.7% according to our model and 2.1% based on the Southlake data.

**Figure 4. One-way Sensitivity Analysis – Health State Utilities**

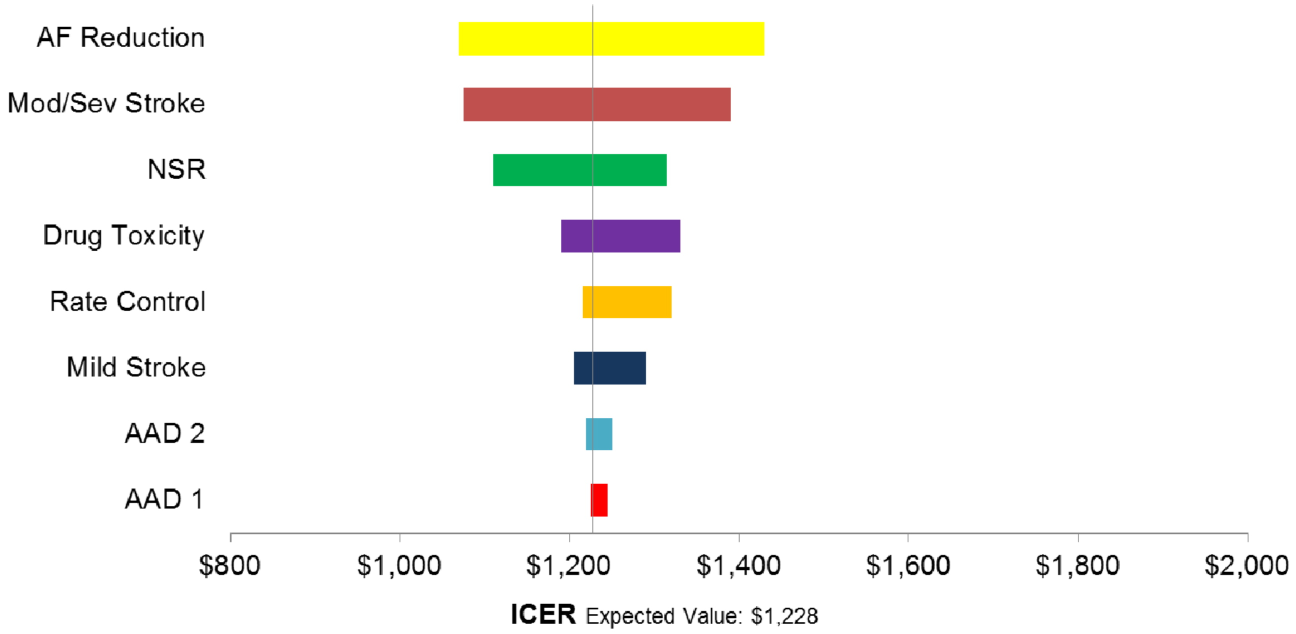

AAD 1: First-line anti-arrhythmic drug therapy; AAD 2: Second-line anti-arrhythmic drug therapy; ICER: Incremental cost-effectiveness ratio; NSR: Normal sinus rhythm

The tornado diagram displays the results of one-way sensitivity analyses for key health state parameters on the ICER for Ablation compared to AAD. The base case ICER is denoted by the vertical line, and changes in the ICER for each parameter are denoted by the horizontal bar.

**Figure 5. One-way Sensitivity Analysis – Ablation Procedure Complications**

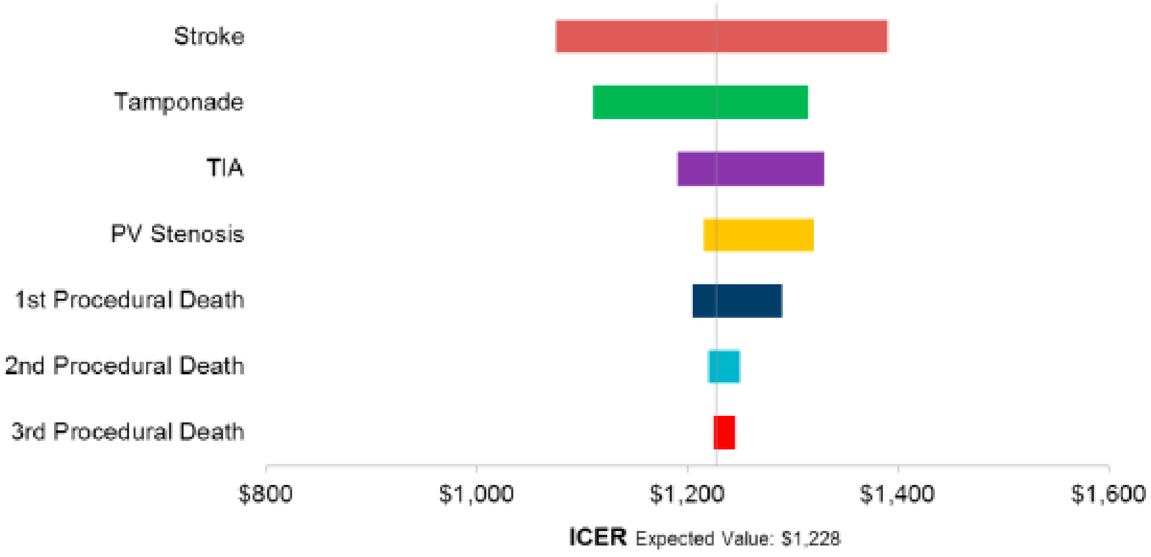

ICER: Incremental cost-effectiveness ratio

The tornado diagram displays the results of one-way sensitivity analyses for ablation complication parameters on the ICER for Ablation compared to AAD. The base case ICER is denoted by the vertical line, and changes in the ICER for each parameter are denoted by the horizontal bar.

## DISCUSSION

This study suggests that CA is a cost-effective strategy when examining a time horizon beyond 5 years. This finding is consistent with previous studies that have generally found CA to be cost-effective compared to AAD when a 5-year or longer time horizon was considered.[8, 18-21] The more favorable results for CA with a longer time horizon reflect that CA treatment involves higher initial costs, while AAD therapy requires treatment costs over a longer time period. These studies all used a payer perspective in their analysis. However, they relied on different clinical trial data for efficacy results, had differing model structures, and were based on practices in different countries. Our study, using real-world outcome data adds to the growing body of literature supporting the cost-effectiveness of CA.

In 2010, the CADTH issued a report that included a cost-effectiveness analysis of CA versus AAD from the Canadian perspective. The CADTH study was based on the model framework of McKenna et al.[21] and found that the ICER of CA compared to AAD was $59,194 per QALY given a 5-year time horizon. Longer follow-up periods made CA considerably more cost-effective.

Moreover, the CADTH systematic review of economic evaluations of CA[8] concluded that existing studies (including their study) examined populations that may not be representative of real-world situations, due to various exclusions and the short-term nature of the clinical trials that formed the basis of these analyses. In contrast, our study used real-world data from a large regional center with longer term follow-up data, mitigating concerns about generalizability.

A comparison of our study with the CADTH analysis[8], the major extant Canadian economic evaluation of ablation, highlights the value of performing additional economic evaluations of catheter ablation using alternative approaches. Our analysis reached similar conclusions, yet with very different sets of assumptions. In contrast to the CADTH analysis, our study: used a Markov modeling approach throughout, whereas CADTH was deterministic in the first year; allowed patients to have up to three ablations (which extended the treatment failure profile of this technology), while CADTH allowed fewer; had a target age of 55 and a $CHADS_2$ score of 0 or 1, instead of 65 and a $CHADS_2$ score of 2 in the CADTH model; and employed data from a large regional center that is more reflective of real-world outcomes, whereas the CADTH study was derived from the McKenna et al. model[21] from the United Kingdom.

Despite these significant differences, a comparison of our model with the CADTH study[8] reveals consistent results with respect to the time horizon needed for CA to be considered cost-effective. Assuming a 5-year time horizon, the ICER results were $59,194 / QALY in CADTH compared to $63,647 / QALY in our model. The ICER results for a 10-year time horizon were $14,273 / QALY in CADTH and $22,879 / QALY in the present study. The greater differences between the 10-year results were largely attributed to a higher number of CA procedures permitted in our model. Because CADTH did not include a lifetime horizon, no comparison for this case can be made. Both studies conclude that CA is a cost-effective treatment alternative to AAD.

Several other studies have examined the cost-effectiveness of CA. Two US-centered studies found CA to be a cost-effective treatment over a lifetime horizon.[18,19] Chan et al.[18] considered a group of 65 year-old patients at low-to-moderate risk of stroke. Employing a lifelong time horizon, that study concluded that catheter ablation was cost-effective, with a cost per QALY of $29,068 for a patient at moderate risk of stroke and $12,134 for a patient at low risk. Reynolds et al.[19] report that the incremental cost-effectiveness of catheter ablation versus AAD was $51,431 after 5 years. This finding was quite sensitive to the time horizon, and for a lifetime horizon, it was highly cost-effective. In addition to time horizon, the results were sensitive to the cost of ablation and patients' quality of life.

In Europe, one study concluded that CA reduced the costs and improved patient outcomes as measured by QALYs.[20] This study was conducted from a Swedish perspective in patients with paroxysmal or persistent AF who had failed on AAD treatment. Sensitivity analysis revealed that ablation remained cost-effective, though no longer always dominant. In a study from the perspective of the United Kingdom National Health Service, McKenna et al.[21] examined the cost-effectiveness of catheter ablation to AAD in AF patients who were refractory to at least one AAD. This study found that ablation was cost-effective over 5-year and lifelong time horizons.

Despite the generally positive results of our analysis and past cost-effectiveness studies, a systematic review of studies on the cost-effectiveness of CA[8,18-23] concluded that there is currently insufficient evidence for drawing firm conclusions about the cost-effectiveness of this treatment option.[12] The study noted that existing research has been limited by a lack of long-term data on the impact of quality of life, stroke risk, and real-world data on ablation procedures. While our study does not introduce new long-term data on stroke risk and quality of life impacts of CA, it is the first to use real-world ablation procedural data, thus providing a better sense of the economic and clinical value of this procedure in actual practice.

Further, economic evaluations of CA using different modeling approaches, treatment patterns, and patient populations, have reached similar conclusions thus confirming the robustness of the findings. Such evidence will improve confidence in CA by alleviating concerns that the results are the artifact of a particular study or approach. This study adds to the growing body of evidence pointing to the cost-effectiveness of CA, and mitigates one of the noted limitations of past studies by incorporating real-world data into the analysis.

### Study Limitations

This study has some important limitations that must be noted. First, the study results pertain to low risk ($CHADS_2$ = 0-1) paroxysmal AF patients from a single center in Ontario, Canada. They may not be generalized to other types of AF patients located in different geographic areas. Other AF patients may differ in demographic and clinical characteristics, ablation techniques used on them, as well as clinical outcomes and concomitant drug therapies. Second, while the model attempted to capture key features of treatments and costs encountered in managing these patients, some simplifying assumptions had to be made, both to render the analysis manageable and in recognition of data limitations. For example, patients who had a stroke were assumed to remain in a stroke state without additional treatment for their AF and patients who suffer drug toxicity from an AAD therapy were assumed to remain in a drug toxicity state without additional treatment. Procedural complication rates were assumed to remain the same for the procedure regardless of patient age (i.e. a 55-year old had the same complication probabilities as a 65-year old). Third, there is a lack of long-term evidence on quality of life for ablation patients as well as long-term stroke data on these patients. Thus, we had to make assumptions about these long-term effects and rely on sensitivity analysis to gauge the robustness of our findings. Despite these limitations, this study contributes to the literature through the use of real-world outcomes data.

## CONCLUSIONS

From the perspective of the Canadian Healthcare system, CA in drug-refractory paroxysmal AF is a potentially cost-effective treatment compared to AAD therapy when examining a time horizon greater than 5 years using real-world outcomes data.

## ACKNOWLEDGEMENTS

The authors would like to thank Jamie March for her thoughts, perspective, and contributions to this study.

### Conflict of Interest Declaration

This study was funded by Biosense Webster, Inc. The publication of study results was not contingent on the sponsor's approval or censorship of the manuscript. Dr. Khaykin and Dr. Verma are affiliated with Southlake Regional Health Center. Ms. Chun and Ms. Olesovsky are employees of Southlake Regional Health Center. Dr. Khaykin and Dr. Reynolds are consultants to Biosense Webster, Inc. Dr. Verma, Ms. Chun and Ms. Olesovsky have no conflicts. Dr. Rizzo is an academic affiliate to and Dr. Mallow is an employee of CTI Clinical Trial and Consulting Services, Inc. CTI is a consultant to Biosense Webster, Inc.
